# 2514. Single Ascending Dose Safety, Tolerability, and Pharmacokinetics of Subcutaneous and Intramuscular CD388, a Novel Long-acting Drug-Fc Conjugate for Universal Prevention of Seasonal and Pandemic Influenza

**DOI:** 10.1093/ofid/ofad500.2132

**Published:** 2023-11-27

**Authors:** Shawn Flanagan, Voon Ong, Tristan Baguet, Roxana E Rojas, Sy-Shi Wang, Ozlem Equils

**Affiliations:** Cidara Therapeutics, San Diego, California; Cidara Therapeutics, San Diego, California; Janssen R&D, Steenhuffel, Vlaams-Brabant, Belgium; Janssen Research & Development, Brisbane, California; Janssen Pharmaceutical, Brisbane, California; Cidara Therapeutics, San Diego, California

## Abstract

**Background:**

CD388 is a novel antiviral drug-Fc conjugate (DFC) designed to deliver universal prevention of seasonal and pandemic influenza. Nonclinical studies have shown CD388 was effective prophylactically against a wide range of seasonal and pandemic influenza A and B strains in lethal and nonlethal mouse models of infection.

**Methods:**

The design of the ongoing blinded CD388 First in Human study, which is being conducted in healthy subjects is shown in Figure 1. CD388 for injection (100 mg/mL) was administered by intramuscular (IM) or subcutaneous (SQ) routes of administration. Single dose pharmacokinetics were determined for subjects in the 50 and 150 mg cohorts of each dosing route at protocol specified nominal time points; alias subject numbers were used to maintain blind. Plasma CD388 concentrations were determined for subjects randomized to receive CD388 using a validated hybrid immunoassay LC-MS method with lower limit of quantitation (LLOQ) of 0.1 µg/mL.

**Figure:**
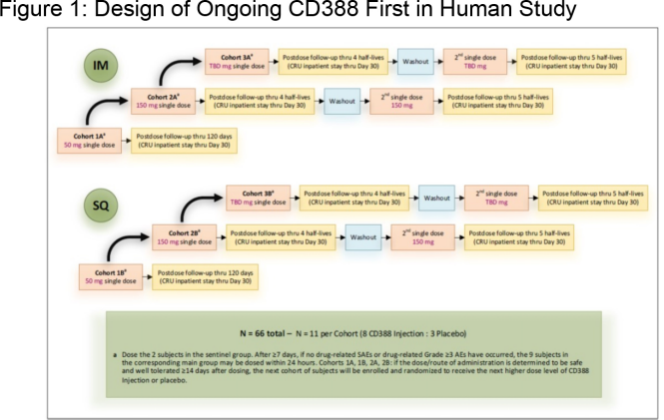

**Results:**

CD388 appeared to be well absorbed by IM or SQ routes of administration with maximum plasma concentrations (T_max_) appearing from 2 to 13 days post dose, and quantifiable concentrations ( >LLOQ) lasting for several weeks to months (Figures 2 to 4). Mean apparent half-life of elimination was ∼ 50 days. Increases in CD388 exposures were approximately dose proportional for both routes (Figures 2 to 4).

No dose limiting local or systemic safety concerns were noted from review of ongoing blinded safety data and the study is proceeding at doses higher than those reported herein.

**Figure:**
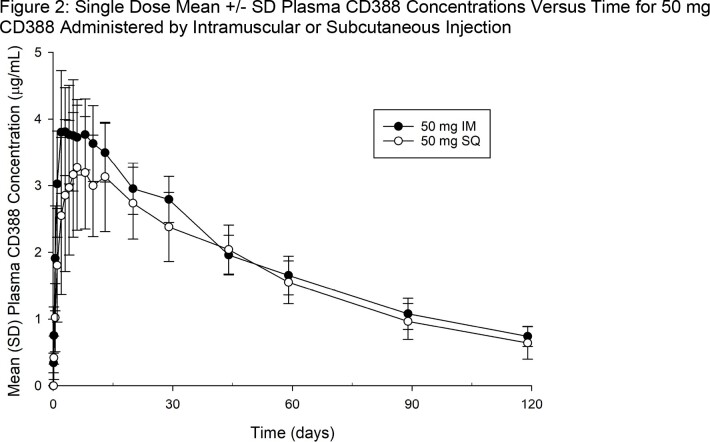

**Figure:**
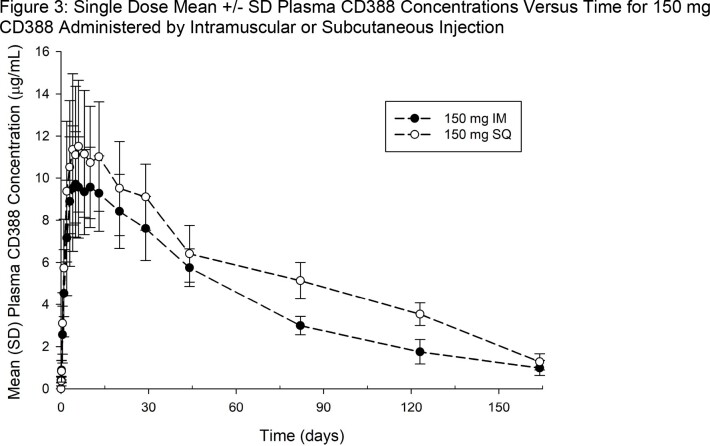

**Figure:**
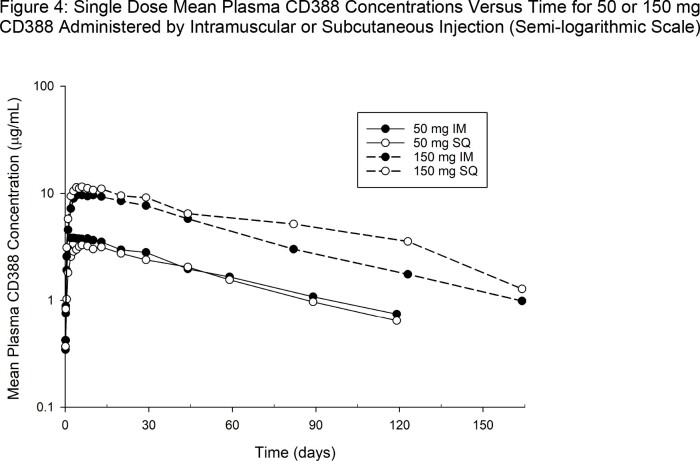

**Conclusion:**

Plasma concentrations of CD388, were similar following intramuscular or subcutaneous routes of administration and were sustained. No safety concerns have been noted in this ongoing study.

**Disclosures:**

**Shawn Flanagan, PhD**, Cidara Therapeutics: Salaried Employee|Cidara Therapeutics: Stocks/Bonds **Voon Ong, Executive Director**, Cidara Therapeutics: Stocks/Bonds **Tristan Baguet, PharmD**, Johnson & Johnson: Employee **Roxana E. Rojas, M.D., Ph.D.**, Janssen: Employee|Janssen: Stocks/Bonds **Sy-Shi Wang, Ph.D.**, Janssen Pharmaceutical: Employee **Ozlem Equils, MD**, Cidara Therapeutics: Employee|Cidara Therapeutics: Stocks/Bonds

